# Frequency and risk factors of pressure injuries in clinical settings of affiliated to Tabriz University of Medical Sciences

**DOI:** 10.1002/nop2.685

**Published:** 2020-11-15

**Authors:** AhmadMirza Aghazadeh, Mojgan Lotfi, Hossein Asgarpour, Mohammad Khajehgoodari, Afsaneh Nobakht

**Affiliations:** ^1^ Department of Basic Sciences Paramedical Faculty Tabriz University of Medical Sciences Tabriz Iran; ^2^ Department of Medical Surgical Nursing Faculty of Nursing and Midwifery Sina Hospital Tabriz University of Medical Sciences Tabriz Iran; ^3^ Department of Surgical Nursing Faculty of Health Sciences Çanakkale Onsekiz Mart University Canakkale Turkey; ^4^ Department of Medical Surgical Nursing Faculty of Nursing and Midwifery Tabriz University of Medical Sciences Tabriz Iran; ^5^ Faculty of Nursing and Midwifery Sina Hospital Tabriz University of Medical Sciences Tabriz Iran

**Keywords:** Braden scale, nurses, nursing, pressure injuries, risk factors

## Abstract

**Aim:**

Pressure injuries are considered a common and costly problem in the care of patients. Prevention and identification of risk factors for pressure injuries are very important due to the high cost of treatment and the adverse consequences of pressure injuries. This study aimed to assess the prevalence of pressure injuries and its risk factors in clinical settings of affiliated to Tabriz University of Medical Sciences.

**Design:**

A descriptive‐analytical study.

**Methods:**

This study was performed on 200 patients who were selected by random sampling. The data collection tool was a 3‐part questionnaire. Data were analysed using a *t* test, chi‐square, Fisher's exact test and logistic regression in SPSS v. 24.

**Results:**

The mean age of the participants was 51.93 (*SD* 14.99) years. The rate of pressure injuries in this study was 19.5%. The most susceptible area for pressure injuries were sacral (35.89%) and gluteal (20.51%), respectively. The pressure injuries was significantly associated with Braden's criteria, age, disease diagnosis and length of hospital stay (*p* < .05). But there was no statistically significant difference between sex and incidence of pressure injuries (*p* > .05).

## 1 INTRODUCTION

Pressure injuries are considered a common and costly problem in the care of patients. The incidence of pressure injuries is a key indicator in nursing care and as a major clinical problem in healthcare delivery (Kottner et al., 2019; Whitty et al., 2017).

The Wound Society defines pressure injuries as follows: “A pressure injury is a localized injury to the skin and/or underlying tissue, usually over a bony prominence, resulting from sustained pressure (including pressure associated with shear).” These injury vary in size and severity of the tissue layers involved, ranging from cutaneous erythema to muscle and bone damage (Stinson et al., 2018).

Pressure injuries often occur in the bones tubercle such as the sacrum, ischial, heel, trochanter, posterior region and scapula (Hu, 2020). The main groups at risk for pressure injuries include patients with spinal cord injuries, the elderly and patients admitted to the intensive care unit (Kula & GaPUD, 2018).

The prevalence of pressure injuries varies depending on the clinical condition. The prevalence of pressure injuries was reported to be 13.6% for acute care patients and 42.1% for long‐term care. The prevalence of pressure injuries in the United States is 14%–17%, in European countries 18.1% and in Iran 19%, which is 5% in the general wards and 10%–21.1% in the intensive care units (Akhkand et al., 2020; Coyer et al., 2017; Karimian et al., 2016; Rafiei, 2016).

The number of patients with pressure injuries continues to rise despite the progress that has been made in early detection and treatment of pressure injuries and international guidelines for wound healing and improved quality of healthcare delivery. Therefore, pressure injuries are a very serious problem worldwide (Jackson et al., 2016).

Patients, families, healthcare centres and the community are significantly affected by the physical, social and economic consequences of pressure injuries (Lotfi, Aghazadeh, Asgarpour, & Nobakht, 2019) that includes the increased burden of care, imposing enormous costs on the health system, decreasing quality of life, increasing pain and risk of infection, delaying recovery, increased frequency and length of hospitalization as well as increased patients’ mortality (Bereded et al., 2018).

In this regard, the most important goal is preventing pressure injuries and identifying the risk factors associated with this complication. To achieve this goal, it is necessary to use of an appropriate tool and the provision of proper nursing care (Lotfi, Zamanzadeh, Valizadeh, & Khajehgoodari, 2019). High sensitivity, good predictive value and easy application in practice are useful features of a pressure injury prediction tool.

Today, there are at least 40 risk assessment criteria, of which only six have been evaluated in terms of validity. Braden Scale is one of these tools that consider the basic dimensions of pressure injuries, including the cause and severity of the wound, as well as tissue tolerance to pressure. Due to the complexity of the process of creating pressure injuries, the need to use a valid objective criterion is routinely raised in hospitals (Wei et al., 2020).

Patients at risk for pressure injuries have been identified and receive more care from caregivers by using this tool in hospitals. As a result, the incidence of pressure injuries and its adverse consequences are reduced (Wei et al., 2020). To plan for the prevention of pressure injuries, this study aimed to evaluate the extent of pressure injuries and determine its risk factors in the Teaching hospitals of Tabriz University of medical sciences.

## 2 METHODS

### 2.1 Design

This study was a descriptive cross‐sectional study carried out in the form of a research proposal approved by Tabriz University of Medical Sciences from November 2018–February 2019.

### 2.2 Setting and participants

The population consisted of all patients (1,057 people) in the internal medicine, surgical and specialist departments of four educational hospitals (Sina, Shohada, Imam Reza and Shahid Madani) of Tabriz University of Medical Sciences, among whom 200 were selected as the sample size. Participants gave informed consent after explaining the study's objectives.

To calculate the sample size, using the following sample size determination equation assuming *Z*: 1.96, *p*: .19, *q*: .81 and *n*: 1,057, the sample size of 194 was required and with counting 2.5% attrition rate of samples, 200 people were selected. Simple random sampling was used for this purpose. Sampling was done according to the available list of beds hospitals in proportion to each ward.

Criteria for inclusion the study included patients admitted to the internal medicine, surgical and intensive care units, the desire to participate in the study, lack of pressure injuries or vascular wounds at the time of hospital admission and patients have been hospitalized for at least 5 days in the aforementioned wards.

Patients' unwillingness to cooperate in research and relocation of eligible patients from the research environment was included exclusion criteria for this study. To collect information, a three‐part questionnaire was used. The first part of the questionnaire was demographic characteristics that including age, sex, weight, length of hospitalization, disease (heart disease, diabetes, limb paralysis or plegia, stroke and spinal trauma), history of major surgery and history of smoking. The second part of the questionnaire was the assessment of pressure injuries risk factors in six dimensions of sensory perception, physical activity and skin moisture status, ability to change position, nutritional status and friction/abrasion using the Braden Scale. Each dimension of the questionnaire has four scores with a Likert form the highest risk to the least risk of pressure injuries except friction and abrasion, which has three scores. The sensitivity and specificity of this scale at the diagnostic point of 18.5 is 92% and its characteristic is 74%. In the patient examination, scores on the Braden scale are divided as follows: score less than nine (very high risk of pressure injuries), score 10–12 (high risk), score 13–14 (moderate risk), score 15–18 (moderate risk) and score more than 19 (safe) (Fazel et al., 2018).

The third part of the questionnaire was skin examination to determine the presence of the pressure injuries, the location of the pressure injuries and the degree of the pressure injuries. All patients were monitored for the incidence of pressure injuries ulcer location and pressure injuries risk factors.

The data were reviewed by the researcher in the morning and evening shifts from the time of admission to discharge during the period of 3 months from April to June 2017. All of these patients were monitored and observed regularly for pressure ulcers, wound sites and risk factors based on the incidence scale. Some information was obtained through the records of patients and others such as skin examination through direct observation and examination of the patient.

The content validity of the questionnaire was confirmed by a panel of experts consisting of eight faculty members of Tabriz University of Medical Sciences. Some minor changes were applied according to experts' recommendations. To determine the reliability of the data collection questionnaire, 15 patients were evaluated independently by two researchers and the correlation coefficient the scores was *r* = .83 (File S1).

### 2.3 Statistical analysis

Data were analysed by descriptive and analytical statistics using SPSS 24 software. Data were summarized using mean and standard deviation for quantitative variables, frequency (%) for qualitative variables; and chi‐square tests and binary logistic regression were used to determine relationship and predictive between variables. All tests were two‐sided, and statistical significance level was set at .05.

## 3 RESULTS

Over the 3‐month study period, 200 patients participated in the study with informed consent. Of these, 117 (58.5%) patients were female and 83 (41.5%) patients were male. The mean age of the patients was 51.93 (*SD* 14.99) years. A total of 39 patients (19.5%) had pressure injuries. No significant relationship was found between pressure injuries and gender (*p* > .05). The results showed that there is a statistically significant direct relationship between age and rate of pressure injuries (*p* < .001) (Table 1). Of the 39 patients with pressure injuries, most had grade II 19 (49%) and grade I 13 (33%). In terms of ulcer location, most ulcers were in the sacral region 14 (35.89%) patients and 10 (25.65%) patients had multiple ulcers in the forearm, waist, head back, ear and knee areas (Table 2).

**TABLE 1:** Variables crosstabs for pressure injury by age and sex

Variable		Pressure injury		Total	χ^2^ test
		Yes	No		
Sex	Female	21 (10.5)	96 (48)	117 (58.5)	χ^2^ = 0.432
	Male	18 (9)	65 (32.5)	83 (41.5)	*p *= .511
					*df* = 1
Age	18–40	0 (0)	46 (23)	46 (23)	χ^2^ = 193.68
	41–60	1 (0.5)	115 (57.5)	116 (58)	*p *= .001
	61–97	38 (19)	0 (0)	38 (19)	*df* = 2

**TABLE 2:** Frequency distribution of pressure injury by depth and location of ulcer

Variables		Frequency	Percent
Depth of ulcer	Grade I	13	33.33
	Grade II	19	48.71
	Grade III	1	2.56
	Grade IV	2	5.14
	Grade I, II	1	2.56
	Grade II, III	2	5.14
	Grade III, IV	1	2.56
	Total	39	100
Location of ulcer	Botex	8	20.51
	Sacrum	14	35.89
	Trochanter	1	2.57
	Multiple areas	10	25.65
	Heel	6	15.38
	Total	39	100

Frequency Distribution Based on Braden Score of 200 patients under study, most patients 141 (70.5%) were in the safe or low‐risk group. Also, out of 39 patients with pressure injuries, 26 patients (67%) were at high risk. The findings of the study showed a significant relationship between Braden score and the incidence of pressure injuries based on the chi‐square test. In other words, with increasing the score of the Braden Scale, the chance of having a pressure injury decreases (Table 3).

**TABLE 3:** Distribution of pressure injury according to Braden score

Braden scale	Pressure ulcer		Total	χ^2^ test
	Yes	No		
Safe (19–23)	1 (0.5)	97 (48.5)	98	χ^2^ = 64.975
Low risk (15–18)	8 (4)	35 (17.5)	43	*p*=.001
Medium risk (13–14)	4 (2)	8 (4)	12	*df* = 4
High risk (10–12)	9 (4.5)	12 (6)	21	
Very high risk (6–9)	17 (8.5)	9 (4.5)	26	
Total	39 (19.5)	161 (80.5)	200	

The incidence of pressure injuries was higher in patients with immobility due to fracture, spinal trauma, head injury, spinal cord injury and stroke than patients with heart failure, renal or gastrointestinal disorders and cancer patients. There was a significant relationship between pressure injuries and type of disease (*p* < .05) (Table 4).

**TABLE 4:** Frequency distribution of pressure injury by type of disease

Diagnosis	Pressure injury		Total	χ^2^ test
	Yes	No		
Fracture, spinal trauma, head injury, spinal cord injury and stroke	30 (15)	32 (16)	62	χ^2^ = 48.18
				*p*=.001
				*df* = 3
HF, HTN, DM, Cardiovascular disease	7 (3.5)	81 (40.5)	88	
Renal or Intestinal disorder	1 (0.5)	34 (17)	35	
Cancer	1 (0.5)	14 (7)	15	
Total	39 (19.5)	16 (1)	200	

Abbreviations: HF, Heart failure; HTN, Hypertension; DM, Diabetes mellitus.

Logistic regression was used to evaluate the relationship between independent clinical variables and the dependent variable of pressure injuries formation due to the duality of this variable. The suitability of this model was confirmed by Hosmer‐Lomeshow test (*p* < .0001). After entering the indicators into the regression model and adjusting the chance ratio, three variables of Braden scale (*p* = .036), hospital duration (*p* = .012) and patient weight (*p* = .014) with the incidence of pressure injuries showed a statistically significant relationship and these variables were considered predictive in this study. As can be seen, increasing the score Braden scale decreases the likelihood that the patient will develop pressure injuries.

Hosmer and Lomeshow (Fagerland & Hosmer, 2012) suggested the chi‐square static which is shown in logistic regression. In order that the model efficient this chi‐square static should be insignificant so the *p* value associated with chi‐square should be greater than .05. This is a different subject with a relation between age groups and pressure injuries.

The share of independent variables including length of hospital stay and weight was almost the same and with one day of hospitalization and one kilogram of weight increased; the probability of the patient getting pressure injuries increased and was equal to .393 and .389, respectively (Table 5).

**TABLE 5:** Results of correlation of Braden scale, length of hospitalization, weight and developing pressure injury variables using regression model

Variables	Coefficient of variation	*df*	*p* value	Odds ratio	Confidence interval for odds ratio	
Braden scale	0.985	1	.03	0.373	0.149	0.936
Length of hospitalization	0.393	1	.01	1.481	1.091	2.012
Weight	0.389	1	.01	1.476	1.011	2.012

The mean length of stay in the hospital was 23.10 (*SD* 21.34) days, and most patients under study were hospitalized within 6–30 days. There was a significant relationship between the length of hospital stay and the pressure injuries (*p* < .05). In other words, by increasing the day length of hospital stay, the risk of pressure injuries increases. There was a direct and significant relationship between the weight of patients and pressure injuries (*p* < .05). That is, as the weight increased, the risk of pressure injuries increased (Table 5).

Results showed that 51% and 1.1% of patients with wet skin and dry skin had pressure injuries, respectively. The incidence of pressure injuries was 10% in patients with a severe decrease in consciousness and sensory perception. This rate was 5.5% in patients with perfectly normal sensory perception. Pressure injuries was observed in 14% of patients with completely immobilized, 4% of patients moving in a chair and 1% of patients with short‐term activity who often spend time in bed. Pressure injuries were also observed in 15% of patients with poor nutritional status or nothing per oral (NPO); however, only 4.5% of patients with adequate nutrition had pressure injuries. These findings were statistically significant (*p* < .05). Table 6 shows the dimensions of the Braden scale and the incidence of pressure injuries.

**TABLE 6:** Mean and standard deviation of Braden scale dimensions in two groups with and without a pressure injury

Dimension	Sensory perception	Moisture	Activity	Mobility	Nutrition	Friction and shear	Total score
Yes	2.69 ± 0.9	2.31 ± 0.2	1.38 ± 0.5	1.67 ± 0.5	2.28 ± 0.5	1.44 ± 0.3	11.72 ± 3.6
No	3.73 ± 0.6	3.38 ± 0.5	2.93 ± 1.5	3.22 ± 1	3.37 ± 0.8	2.43 ± 0.5	18.98 ± 4.6
*p* value	*p* < .002	*p* < .005	*p* < .003	*p* < .005	*p* < .005	*p* < .005	*p* < .001

The findings also show that 18.5% of patients who had problems with friction and shear had bed sores. Only 1% of patients who did not have friction problems had bed sores. Chi‐square test showed a significant relationship between these two variables (*p* = .001) (Table 6). There was a significant relationship between pressure injuries and type of inpatient ward (*p* < .003), urinary and stool incontinence (*p* < .001) and history of major surgeries (*p* < .001).

## 4 DISCUSSION

Despite the progress and measures that have been taken in the field of preventing the occurrence of pressure injuries, however, not all the causes of pressure injuries are known. In fact, there is no single factor for the occurrence of pressure ulcers and the interaction of many risk factors increases the risk of developing pressure injuries (Serrano et al., 2017).

According to the findings of the present study, the incidence of pressure injuries was 19.5%., the incidence of pressure injuries in internal‐surgical wards is 8.5% and in ICU is 11%. In the intensive care unit, due to long‐term hospitalization, patients' deteriorating condition and decreased level of consciousness, the prevalence of pressure ulcers is higher, which is consistent with the results of a study by Sohrabi et al. (Akhkand et al., 2020). Studies show that approximately 60% of pressure injuries occur in the first two weeks of hospitalization in the intensive care unit (Pachá, Faria, Oliveira, & Beccaria, 2018).

In this study, no significant relationship was found between pressure injuries and sex as in other studies (Lichterfeld‐Kottner et al., 2020; Pachá et al., 2018). In our study, it was found that increasing age had a significant effect on pressure injuries and more than 90% of pressure injuries were reported in patients over 60 years of age that the main reason for this issue is due to less mobility and activity of older people.

Elderly people seem to have wrinkled skin due to subcutaneous fat loss and are prone to pressure injuries. Studies show that 66% of older people admitted for orthopaedic surgery are at risk for pressure injuries (Amirifar et al., 2013).

The results of our study showed that with a one‐day extension to the length of hospital stay, the risk of developing pressure injuries increases. All texts agree on the direct effect of length of stay on pressure injuries. Hospital stay has the greatest impact on pressure injuries, regardless of other causes of developing pressure injuries especially in the intensive care unit due to the limitation of the patient's movement and activity.

The lyder study also showed that the onset of pressure injuries in a hospital increases the length of hospital stay and the likelihood of readmission. This finding can be considered from two perspectives: (a) wound healing is slow in the third and fourth degrees that caring for it requires increasing the length of hospital stay of patients; and (b) increased inactivity and the presence of pressure ulcer risk factors in individuals with the worsening disease and longer hospital stay may exacerbate the degree of pressure injuries (Lyder et al., 2012).

According to the results of the study, the diagnosis of the disease is involved in the development of pressure injuries. In this study, most patients with pressure injuries were hospitalized with a diagnosis of fracture, spinal trauma and stroke. In fact, the incidence of pressure injuries is higher in patients with limited mobility. Since the main cause of pressure injuries is related to prolonged pressure on the skin and subsequent lack of blood supply to the organ and this has been confirmed in most studies, it seems that immobility can be a predictor of this complication (Ramezanpour et al., 2018; Rashvand et al., 2020; Ueno et al., 2020).

Afkar's found that moisture is a primary risk factor for the development and progression of pressure injuries which was consistent with the findings of our study (Afkar et al., 2014). But in the Kermani Reihani et al. study, moisture was not identified as a risk factor for pressure injuries. They stated that this factor was not recognized as a risk factor due to the use of Foley catheters and low humidity in the patients under study, probably (Reihani & Haghiri, 2007).

In this study, decreased level of consciousness was identified as one of the risk factors for pressure injuries which is similar to the study by Afkar et al. (2014). The results of Cooper's study showed that patients with lower levels of consciousness had less sensory perception due to anaesthesia and sedation drugs and patients could not perceive pain caused by severe pressure or independently change position or request the change position. These two factors increase the incidence of pressure injuries in patients (Cooper, 2013). In the Fallahinia study, the level of consciousness was not recognized as a risk factor for pressure injuries. Due to the fact that despite the high level of consciousness of patients, their mobility was low and so they got pressure injuries (Soltanian, 2013).

As a result of friction and sliding force, the capillaries of the skin and deeper tissues become disrupted and the skin progresses to pressure injuries. The results of our study were consistent with other studies on the increased risk of pressure injuries in patients who have more friction or abrasion at the time of change position (Serrano et al., 2017). But our findings contradict the results of the Kermani Reihani study, this difference can be attributed to differences in the quality of nursing care (Reihani & Haghiri, 2007). While in the Fallahinia study, it was found that most of the patients' caregivers are unfamiliar with the principles and appropriate method of patient transfer (Soltanian, 2013).

Patients with malnutrition often have severe muscle atrophy and a decrease in the subcutaneous tissue. With these changes, there is less tissue to protect between the skin and the bones below it. Therefore, the effect of pressure on the residual tissue intensifies and the risk of pressure injuries increases (Neloska et al., 2016).

In the Serpa study, nutrition was not recognized as a risk factor for pressure injuries and its cause can be attributed to proper nursing care as well as patient nutrition with the opinion of a nutritionist in a planned manner (Serpa et al., 2011). While in the Fallahinia study, patients were left alone at home and most patients with spinal cord lesions had difficulty feeding and swallowing and needed principled and scientific care (Soltanian, 2013).

## 5 CONCLUSION

Pressure injuries are unavoidable in some patients in hospitals. Risk factors of pressure injuries in the studied centres was included old age, immobility caused by fractures and spinal trauma, decreased sensory perception, increased skin moisture, inappropriate nutrition, decreased activity and mobility and increased friction and abrasion.

In the present study, the quality of nursing care in the evaluation of the patient and the risk factors for pressure injuries in most cases has been undesirable. However, risk assessment is recommended as the first measure to prevent pressure injuries in nursing care and identifying patients who are at risk for pressure injuries is critical to effective treatment. It seems that the poor quality of patient evaluation and the risk factors for pressure injuries related to the lack of knowledge of nurses, lack sense of responsibility of nurses and most importantly the lack of a reliable tool to assess the risk of pressure injuries. Therefore, special education of nurses and other hospital staff to care for patients, using a standardized patient assessment method and attention to the causes of pressure injuries can be effective in identifying patients at risk and reducing the incidence of pressure injuries.

## 6 RELEVANCE TO CLINICAL PRACTICE

Based on the results of this study, all dimensions of the Braden scale had a significant relationship with pressure injuries incidence, meaning that the Braden scale is a good tool for pressure injuries prediction. It is suggested that training and use of this tool in inpatient wards be considered and added to patient records if possible.

## CONFLICT OF INTEREST

The authors declare that they have no conflicts of interest.

## AUTHOR CONTRIBUTIONS

All authors (ML, AA, MKH, HA, and AN) have participated in the conception and design of the study. ML contributed the data collection and prepared the first draft of the manuscript. AA, HA and AN, Critically revised and checked the proposal closely, the analysis and interpretation of the data and design the article. All authors read and approved the final manuscript.

## ETHICAL APPROVAL

We obtained institutional review board approval for this study to collect and analyse data from the Committee of Ethics at Tabriz University of Medical Sciences with number IR.TBZMED.REC.1397.53.

## COMPLIANCE WITH ETHICAL STANDARDS

The ethics committee of Tabriz University of medical sciences authorized the permission to conduct this study (Ethical no is IR.TBZMED.REC.1397/53). All of the authors have full control of all primary data, and they agree to allow the journal to review their data if requested.

## Supporting information

File S1

## Data Availability

The data used to support the finding of this study are available from the corresponding author on reasonable request.
